# Comparative Emulsifying Properties of Octenyl Succinic Anhydride (OSA)-Modified Starch: Granular Form vs Dissolved State

**DOI:** 10.1371/journal.pone.0160140

**Published:** 2016-08-01

**Authors:** María Matos, Ali Marefati, Gemma Gutiérrez, Marie Wahlgren, Marilyn Rayner

**Affiliations:** 1 Department of Food Technology, Engineering, and Nutrition, Lund University, P.O. Box 124, SE 221 00 Lund, Sweden; 2 Department of Chemical and Environmental Engineering, University of Oviedo, Julián Clavería 8, 33006 Oviedo, Spain; The University of Tokyo, JAPAN

## Abstract

The emulsifying ability of OSA-modified and native starch in the granular form, in the dissolved state and a combination of both was compared. This study aims to understand mixed systems of particles and dissolved starch with respect to what species dominates at droplet interfaces and how stability is affected by addition of one of the species to already formed emulsions. It was possible to create emulsions with OSA-modified starch isolated from Quinoa as sole emulsifier. Similar droplet sizes were obtained with emulsions prepared at 7% (w/w) oil content using OSA-modified starch in the granular form or molecularly dissolved but large differences were observed regarding stability. Pickering emulsions kept their droplet size constant after one month while emulsions formulated with OSA-modified starch dissolved exhibited coalescence. All emulsions stabilized combining OSA-modified starch in granular form and in solution showed larger mean droplet sizes with no significant differences with respect to the order of addition. These emulsions were unstable due to coalescence regarding presence of free oil. Similar results were obtained when emulsions were prepared by combining OSA-modified granules with native starch in solution. The degree of surface coverage of starch granules was much lower in presence of starch in solution which indicates that OSA-starch is more surface active in the dissolved state than in granular form, although it led to unstable systems compared to starch granule stabilized Pickering emulsions, which demonstrated to be extremely stable.

## Introduction

Formulations based on emulsions are present in many fields, such as processed foods, paints and coatings, personal care products, agro-chemicals and pharmaceuticals, among others. Emulsions are defined as a two-phase system consisting of two immiscible liquids of different composition, one of which is in the shape of droplets, dispersed in the other one. Emulsion droplet stabilization is often achieved through the addition of amphiphilic molecules such as emulsifiers, which decrease the interfacial tension between the phases and increase the steric hindrances and/or the electrostatic repulsion between the droplets [1,2].

The use of particles to stabilize emulsions, instead of surfactants, has received substantial and increased research interest because of their distinctive characteristics and promising technological applications in a range of fields [3–12]. A Pickering emulsion is an emulsion that is stabilized by solid particles which adsorb onto the interface between the two phases.

This special type of emulsion was originally observed independently by Ramsden [13] and Pickering [14].

Properties such as shape, size and hydrophobicity of the particles may affect the stability of the emulsion. Particles should also have partial dual wettability for both phases. The contact angle of the particles to the surface of the droplet is a characteristic of the hydrophobicity. Therefore, particles that are partially hydrophobic (i.e. contact angle of approximately 90°) are better stabilizers because they bind better to the surface of the droplets. This leads to the spontaneous accumulation of particles at the oil-water interface with subsequent stabilization against coalescence by volume exclusion and steric hindrances [15], i.e. the particles prevent oil-water interfaces of oil droplets from coming into direct physical contact. This phenomena is explained by the fact that if the particles have favorable wetting properties (i.e. not too close to zero or 180°) and are above a certain size (approximately 10 nm) their adsorption at the oil water interface is effectively irreversible as the desorption energy per particle is several thousand kT [4,15]. Under these conditions particles show irreversible adsorption, in contrast to surfactant molecules, which exist in a dynamic equilibrium at the oil water interface and can adsorb and desorb on a rapid timescale. This strong adsorption of particles at the interface may also explain their stability (even with large droplet sizes) over extended periods of time [7,16]. Therefore, Pickering emulsions are extremely stable against coalescence and Ostwald ripening and the potential to enhance oxidative stability compared to systems stabilized by surfactants [4,8,15,16].

In previous studies, starch granules isolated from Quinoa were modified with octenyl succinic anhydride (OSA) and used to produce Pickering emulsions with excellent stability [7,9,17]. It was shown that the barrier properties of this type of emulsions were enhanced by heating *in situ* since the surface coverage starch granules can be partially gelatinized creating a more impermeable layer at the droplet surface [7–10,18,19]. The gelatinization process includes swelling of starch granules, amylose leakage from granules, and loss of molecular and crystalline order.

In addition, chemically modified (OSA) potato and barely starches have been previously used in the molecularly dissolved state to stabilize emulsions. Since this type of hydrophobically modified polysaccharides are also of great interest in food formulations due to their amphiphilic character that can be adsorbed at interfaces for stabilization [20,21]. Results indicate that it is possible to create an emulsion with the OSA-starch as the sole emulsifier but so far no studies with OSA-modified starches isolated from Quinoa in solution have been reported.

The purpose of this work was to compare the stability and droplets size of food emulsions using OSA-modified starch from Quinoa in the dissolved state and particulate state as emulsifier. Furthermore, this study aims to understand mixed systems of particles and dissolved starch with respect to what species dominate at the interface and how the stability is affected by addition of one of the species to already formed emulsions.

Emulsions were prepared using OSA-modified starch isolated from Quinoa in the granular form or in solution, using the same range of emulsifier concentrations, as well as mixtures of both forms considering the addition sequence (adding particles or molecules first or second during emulsification). All emulsions were characterized with respect to particle size distribution (PSD), visual microstructure under the light microscope and stability in a Turbiscan Lab apparatus.

## Materials and Methods

Native starch was isolated from Quinoa grains (Chenopodium quinoa Willd.) (Product of Bolivia, purchased from Biofood-Biolivs AB, Sweden) by a wet-milling process and OSA-modified with a degree of substitution of 2.2% using the method described in Rayner et al. 2012 [7]. The external water phase was a 5mM phosphate buffer with 0.2M NaCl, density 1009.6 kg/m^3^, at 20°C. Particles size distribution of both types of starches (native and OSA-modified) ranged from 1 to10 μm as reported in previous studies [9, 17]. Quinoa starch granules are irregular polygonal shaped with smooth edges and have a surface weighted mean diameter or Sauter mean, D_[3,2]_, of approximately 2 μm. The particles were also observed under SEM and it was observed that they were uni-modal but with some aggregates of granules generating the peak at 10 μm [9, 17]. The oil phase was the medium-chain triglyceride oil Miglyol 812, density 945 kg/m^3^ at 20°C (Sasol GmbH, Germany). Miglyol 812 is a mixture of caprylic/capric triglycerides (caprylic acid: C8, capric acid: C10). At room temperature liquid lipid (oil) of low viscosity. Usually, fatty acid composition in medium chain triglycerides is dominated by C8 fatty acids (50 to 65%), followed by C10 (30 to 45%), C12 (max. 5%) and C6 (max. 3%). An oil-soluble dye (oil red EGN (ICN Biomedicals Inc., USA), molecular formula C_25_H_22_N_4O_O) was added to visualize the eventual presence of free oil in the formulated emulsions.

### Preparation of starch Pickering emulsions

Pickering emulsions with a 7% and 33% (w/w) of Miglyol 812 were prepared in 7 mL volumes. The oil phase was dispersed in a continuous phase of 5mM phosphate buffer with pH 7.0 0.2 M NaCl containing varying concentrations of starch from quinoa in granular non-dissolved state. This was emulsified in glass test tubes by high shear mixing by an Ystral X10 mixer (Ystral GmbH, Germany) with 6mm dispersing tool at 22000 rpm for 30 s. Formulation and stirring conditions were based on previous studies [9]. All the emulsions were prepared in triplicate. Sodium benzoate was added to prevent contamination of microorganism under storage.

### Preparation of emulsions stabilized with starch dissolved state

The starch solution samples were prepared by dissolving the starch granules (both the native and the OSA-modified), in 50 mL of buffer 5mM phosphate buffer at a pH 7.0 0.2 M NaCl and then transferring the sample to an autoclave cylinder.

The dissolution method was chosen taking into account previous studies where the maximum dissolution temperature with minimal degradation was determined either for amylose or amylopectin solutions [22,23]. A high-pressure laboratory autoclave model II (Carl Roth GmbH & Co KG, Karlsruhe, Germany) was used, equipped with a tablet magnetic stirrer and programmable temperature control unit (WRX 2000). Before heating, the sample was flushed with nitrogen gas for 5 min to avoid oxidative degradation of the sample during heating. The solution/suspension was then heated from room temperature to 150°C (taking 15 min to heat-up from room temperature). The temperature was then maintained at 150°C for 40 min, and after heating, the autoclave cylinder was quenched to approximately 40°C by immersion in a cold water bath.

Autoclaving at temperatures between 150°C and 155°C were used in other studies for high amylose starch dissolution [24,25]. However, some authors have reported that such conditions might lead to degradation of the starch polymers when experiments were carried out using glycogen as model for amylopectin [22].

As reported in previous studies, samples should be analysed fresh for reliable characterization [23]. Therefore, all emulsions were formulated by dilution from a starch stock solution of 30 mg/mL. The viscosities of the starch solutions used as external phases were determined. Viscosity measurements were carried out in a Mars II rheometer (Haake, Germany) using plate-plate configuration at 25°C at 100 s^-1^ constant shear rate for 180 s^-1^. Viscosities of starch solutions were similar with both types of starches. At low concentrations (4–8 mg/mL) values were in the range 1–1.5 mPas. While at higher concentrations values ranged from 3.5 mPas at 15 mg/ml to 29 mPas at 30 mg/ml getting values up to 2767 at 100 mg/ml.

Emulsions were prepared with the same starch to oil ratios and emulsification conditions as for the Pickering emulsions.

### Preparation of emulsions stabilized by a combination of starch granules and starch dissolved

Emulsions were stabilized by a combination of starch as granules and in solution with a ratio 1:1. Emulsions were prepared at 7% oil (w/w) by simultaneously mixing the starch in granular and molecular form, the continuous phase and the oil phase emulsifying the mixture under the same homogenization conditions as described for Pickering emulsions.

To investigate the effect of different orders of addition of emulsifier, a 33% (w/w) oil content emulsion was first prepared using the starch in one of the physical states (*i*.*e*.: granules (G)) and then re-dispersed in the other continuous phase containing the starch in the other aggregation state (*i*.*e*.: dissolved (D)) resulting in a final oil content of 7% (w/w). The same homogenization conditions aforementioned were used in both steps of emulsification.

These experiments were carried out with the OSA-modified starch granules, but there was also a final set of experiment in which 7% (w/w) oil emulsions were prepared with mixtures of OSA-modified granules and native starch dissolved.

### Emulsion characterization

Particle size distributions of the emulsions were measured by light scattering using a Malvern Particle Size Analyzer (Mastersizer 2000S, Malvern Instruments Ltd. UK). The sample was added to the flow system containing milliQ water and was pumped through the optical chamber at a pump setting of 2000 rpm. A refractive index (RI) of 1.54 was used for the emulsions samples while the RI of the continuous phase was set to 1.33 (water), and the obscuration ranged between 10 and 20%.

Micrographs of the emulsions were obtained with a light microscope (Olympus BX50, Tokyo, Japan) with 10-100x magnification and digital camera (DFK 41AF02, Imaging source, Germany) with the software ImageJ (NIH, version 1.42m) just after emulsification.

Emulsion stability was evaluated using a Turbiscan Lab Expert (Formulaction Co., France) by static multiple light scattering (MLS), which operates by sending a light beam through a cylindrical glass cell containing the sample. Emulsions were placed without dilution in the test cells and the transmitted and backscattered light were monitored as a function of time and cell height at 30°C. The optical reading head scanned the sample in the cell, providing Tansmission (TS) and Backscattering (BS) data every 40 μm as a function of the sample height (in mm). These profiles build up a macroscopic fingerprint of the emulsion at a given time providing useful information about changes in droplet size, appearance of creaming or a clarification processes allowing one to monitor the height of the clarification front and migration velocity of creaming oil droplets as a function of time [26]. Turbiscan equipment has been widely used to select the best formulation for colloidal systems employed in food or cosmetic applications [27–32].

## Results and Discussion

### Emulsions formulated with OSA-modified starch in the granular form or molecularly dissolved at 7% (w/w) oil content

**Fig 1:**
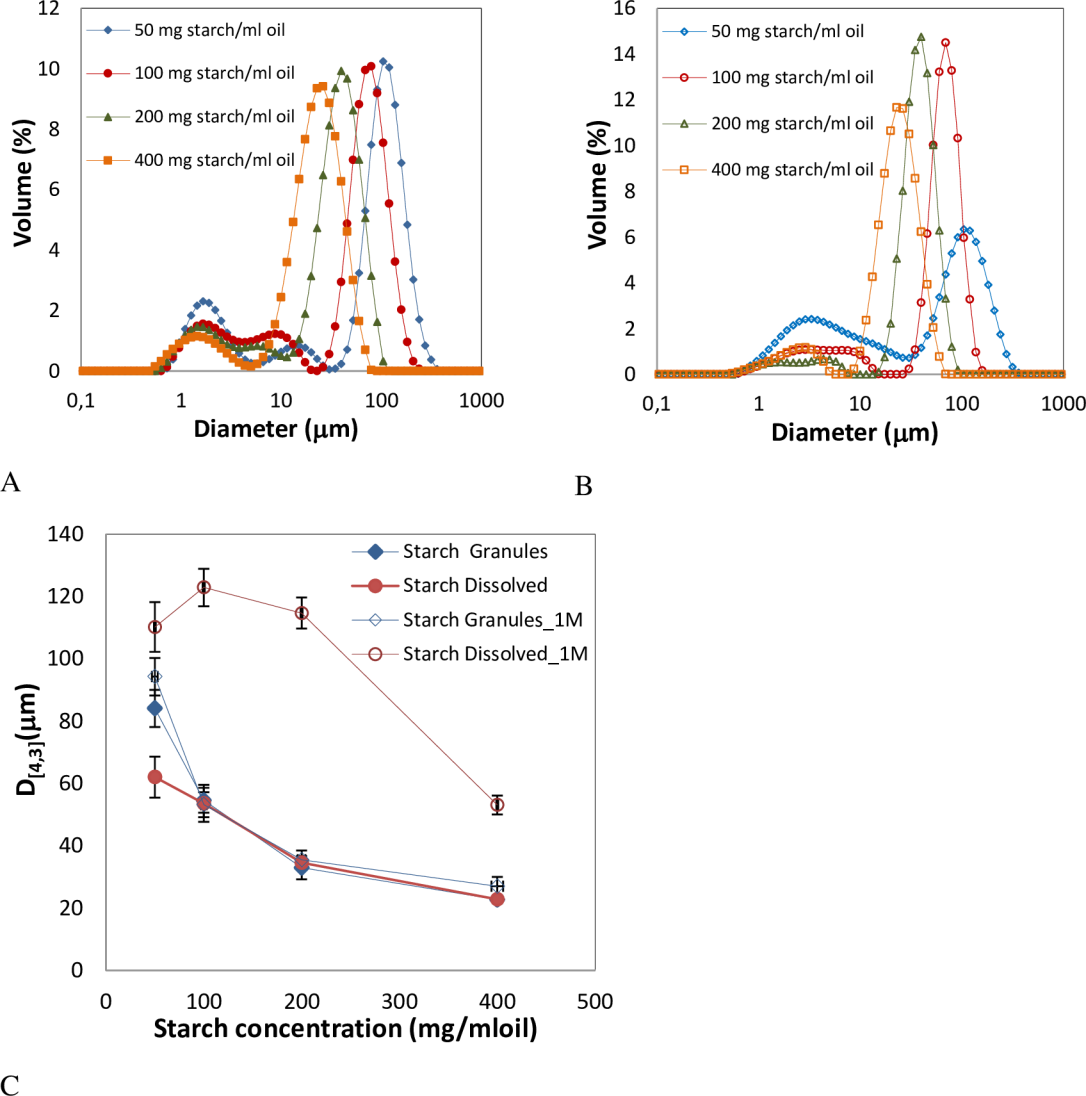
Droplet size distributions of emulsions prepared with 7% (w/w) oil content at different OSA-modified starch concentrations. A) Emulsions stabilized with granules. B) Emulsions stabilized with starch on the dissolved state. C) Mean volume diameter, D_[4,3]_, of emulsions prepared with 7% (w/w) oil content at different OSA-modified starch concentrations stabilized both with granules or starch dissolved. D_[4,3]_, was measured just after preparation and after 1 month under storage (1M).

**Fig 2:**
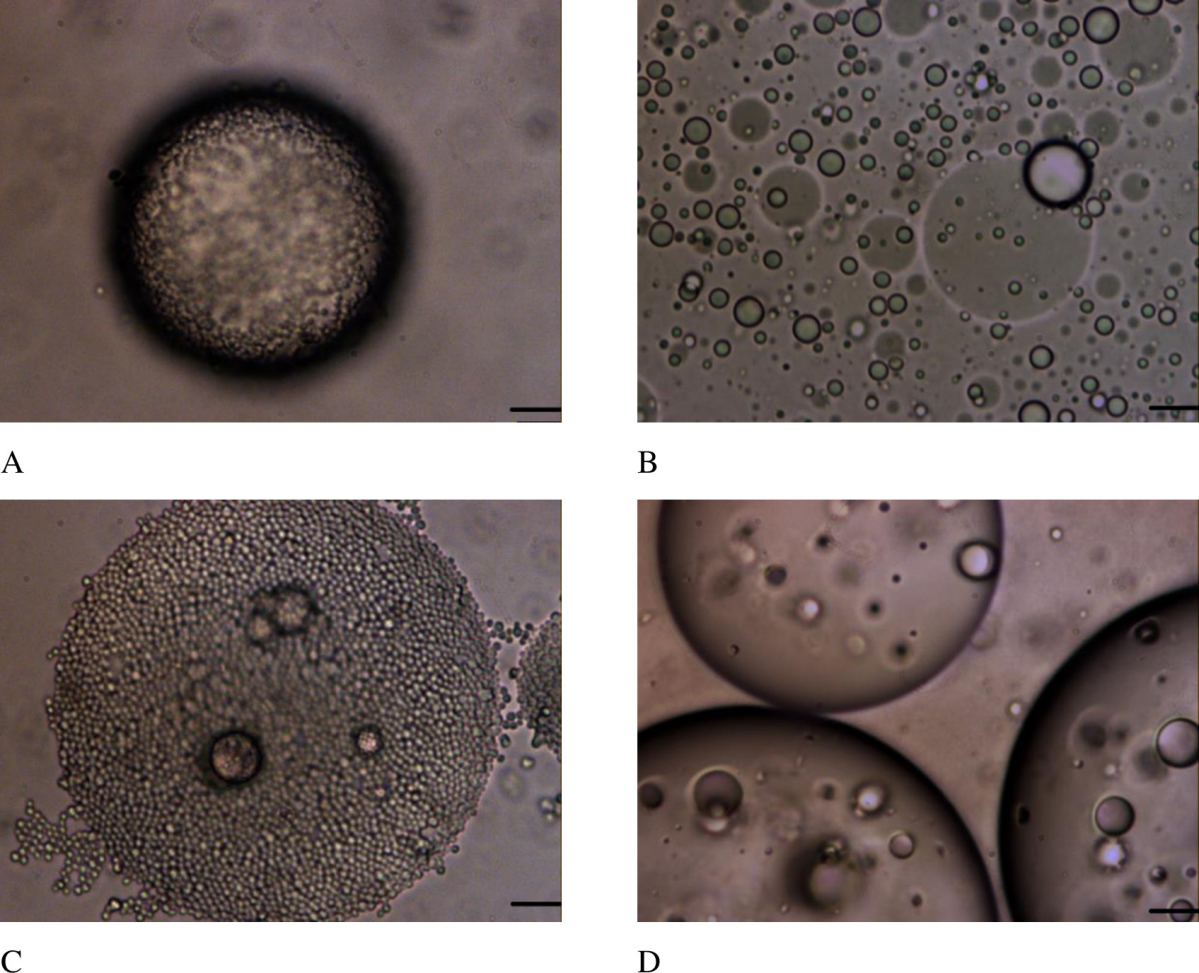
Micrographs of emulsions stabilized with OSA-modified starch granules (A-C) and OSA-modified starch in the dissolved state (B-D). A,B) Emulsions prepared at 7% (w/w) oil and stabilized using 50 mg starch/ml oil. C,D) Emulsions prepared at 33% (w/w) oil and stabilized with 100 mg starch/ml oil. Scale bar: 10 μm.

For emulsions prepared with OSA-modified starch in the granular form or in solution at 7% (w/w) oil, similar droplets sizes were obtained, which decreased as the amount of starch was increased (Fig 1), as expected [6,17]. Slight differences were observed in D_[4,3]_ at 50 mg starch/ml oil (Fig 1C) but high similarity was observed in droplets size distributions exhibited in Fig 1A and Fig 1B. Bimodal droplets size distributions were obtained with sizes in the range ∼25–95 μm being in good agreement with observations under the microscope (Fig 2). The starch granules can be easily identified around the surface of the oil droplets in case of Pickering emulsions (Fig 2A).

Previously, chemically modified (OSA) potato and barely starches in solution have been used to stabilize emulsions and results showed that it was possible to generate an emulsion with OSA-starch as the sole emulsifier since as its high molar mass and branched polymer structure allowed it to be adsorbed at the interface, leading to steric stabilization of emulsions [20,21]. The Sauter mean diameter reported by Nilsson et al. [20] was between 0.4 and 11μm. But in those studies both the dissolution method (boiling water bath during 10 min), the emulsification process (3 minutes at high shear mixer and then high pressure homogenizer at 15 MPa) and the concentration range (7.98–31.92 mg starch per ml oil) were not the same than in this work. Therefore, these differences can affect the adsorption rate leading to different mean droplet sizes.

However, huge discrepancies were observed in the stability of emulsions prepared with OSA-modified starch in the two different forms. While emulsions prepared with starch granules maintained a constant droplet size after one month, indicating high stability, emulsions formulated with dissolved starch were very unstable showing variations up to 113% in particles size between fresh and stored emulsions (Fig 1C). Backscaterring (BS) and Transmission (TS) profiles also confirmed these results.

**Fig 3:**
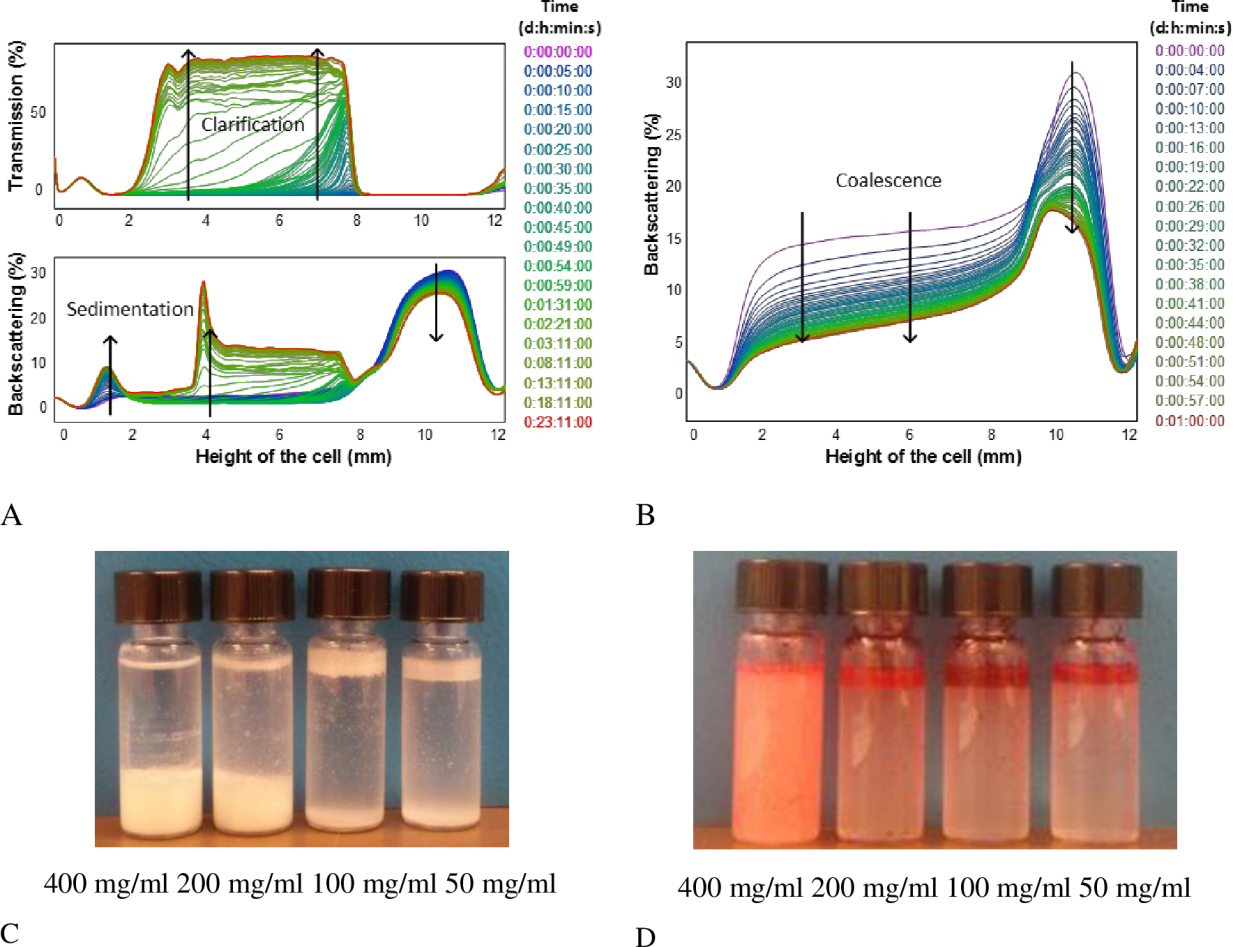
Kinetic ΔBS profiles of a 7% (w/w) oil emulsions. A) Emulsions stabilized with OSA-modified starch granules at a concentration of 50 mg /ml oil. B) Emulsions stabilized with OSA-modified starch on the dissolved state at a concentration of 200 mg /ml oil. C, D) Turbiscan glass cells showing these emulsions respectively aspect after the monitoring time (1h). Note: Oil red EGN was added to visualize the eventual presence of free oil.

All Pickering emulsions were stable for several weeks at room temperature and neither coalescence nor Ostwald ripening was observed showing little change in appearance and emulsions layer height, according to previous results where no variations were observed even after several months [6,9]. An example of the transmission (TS) and backscattering (BS) profiles obtained for Pickering emulsions prepared with OSA-modified starch granules are shown in Fig 3A. Similar profiles were observed for all samples with slight differences in behavior as the starch concentration was varied.

The highest BS values were observed in all cases at the top of the cell (from 8 to 12 mm) indicating the presence of emulsion droplets in this region. A BS decrease was observed with time at the top accompanied with a simultaneous increase at the bottom, promoted by the sedimentation of the unadsorbed granules in the continuous phase or small starch granule stabilized emulsion droplets dense enough to sink due to a large enough starch to oil ratio [6]. This process lasted until the end of the monitoring time and an increase in BS (Fig 3A) at the middle of the cell was observed due to the presence of either starch or emulsion sediment, depending on each case.

No transmitted light was observed along the cell at the beginning of the monitoring time. However, a TS increase was noticed in the middle of the cell (from 2 to 8 mm) while sedimentation was taking place. The thickness of this front changes being smaller for higher starch concentrations since a higher emulsion index was obtained. The % of transmitted light is inversely proportional to the amount of starch added according to the corresponding BS profiles. Larger variations in clarification fronts heights were observed at lower starch concentrations.

For emulsions formulated with higher starch concentrations (200 and 400 mg starch per ml of oil) oil droplets settled at the bottom of the cells (Fig 3C). These differences were reflected in the width of the BS peaks both at the bottom and top of the cell. In the case of emulsions prepared with lower concentrations (50 and 100 mg starch per ml oil) oil droplets remained at the top and very slight presence of sediment was observed at the bottom. This is in good agreement with previous results where it was reported that the droplet size decreases as the amount of starch increases and therefore their density also increases since the relative volume of oil to the starch layer covering it is smaller [6]. Another effect of high starch concentration was that the accumulation of starch granules between droplets increased their total effective density and the resulting emulsion phase [17].

Larger BS variations along the height of the cell were observed in emulsions prepared with dissolved starch. An example of the BS profiles of emulsions prepared with OSA-modified starch in the dissolved state is shown in Fig 3B. A clear decrease of BS over the whole height of the sample was observed, in the case of emulsions formulated with starch concentrations in the range 50–200 mg/ml, an indication that coalescence occurred increasing the emulsion droplet sizes. Fig 3D shows the glass cells after the monitoring process. As can be seen free oil is present (seen red by the oil soluble dye) at the top of the emulsions.

Larger variations in the BS profiles were observed (Table 1) for emulsions prepared with dissolved starch at lower initial starch concentration (0–200 mg starch/ml oil) being in the range 50–72%. But in this case, the BS variations were produced by changes in droplet sizes since it was evident that a coalescence process occurred. Changes in mean diameter were estimated with Turbiscan software assuming a constant oil fraction (ϕ) of 7% within the calculated zone (middle of the cell). Variations from 21 to 195% were observed with no clear trend at low starch concentrations confirming the instability of these emulsions.

**Table 1:** Backscattering variations, ΔBS, changes in diameters, Δd/d_o_, and migration velocities in the middle of the cell for emulsions formulated with 7–33% (w/w) oil content at different concentrations of OSA-modified and native starch dissolved (molecules).

Starch concentration (mg/mloil)	% Oil Content (w/w)	Type of starch	ΔBS (%)	Δd/d_o_ (%)	BS front velocity (μm/s)	TSI
50	7	OSA-modified	54	101	13	9
100	7	OSA-modified	50	76	17	7
200	7	OSA-modified	72	195	8	12
400	7	OSA-modified	16	21	1	6
50	33	OSA-modified	33	43	9	7
100	33	OSA-modified	40	35	9	6
50	7	Native	69	1430	12	9
100	7	Native	70	207	12	15
200	7	Native	60	149	5	15
400	7	Native	10	14	1	6

Adsorbed macromolecules which are soluble in the continuous phase give rise to steric stabilization mainly with entropic contributions but destabilization may occur if the surface coverage is insufficient. It could allow the macromolecules to form bridges between particles and the non-adsorbed macromolecules may be depleted from the vicinity of the surface and can give rise to depletion flocculation [20,33,34].

The BS front velocities of emulsions droplets were obtained from the slope of the phase thickness (delta H) of the clear phases, from BS profiles versus time. They were much higher for emulsions prepared with lower starch concentration, exhibiting values in the range 13 or 17μm/s for 50 or 100 mg/ml oil confirming that destabilization phenomena occurred faster. Emulsions prepared with 400 mg starch per ml oil showed the lowest migration velocity value and the smallest BS variations (16%). This was in good agreement with microscope observations. Larger droplets coalesced fast even forming a continuous thin oily film. This explains the red color observed at the top of the cells after the addition of oil soluble dye (Fig 3D).

Turbiscan stability index (TSI) was also calculated. In order to compute it, the intensity of light toward the complete cell height was compared mathematically, based on a scan-to-scan difference. It sums all the variations detected in the samples in terms of size and/or concentration and is defined by the following equation:

$$TSI=\sum_{i}\frac{\sum_{h}\left|{scan}_{i}-{scan}_{i-1}\right|}{H}$$ (1)

being H the height of the cell.

Variations in the range 6–15% were observed with the lowest variations at the highest starch concentration.

### Emulsions formulated with OSA-modified starch in the granular form or molecularly dissolved at 33% (w/w) oil content

**Fig 4:**
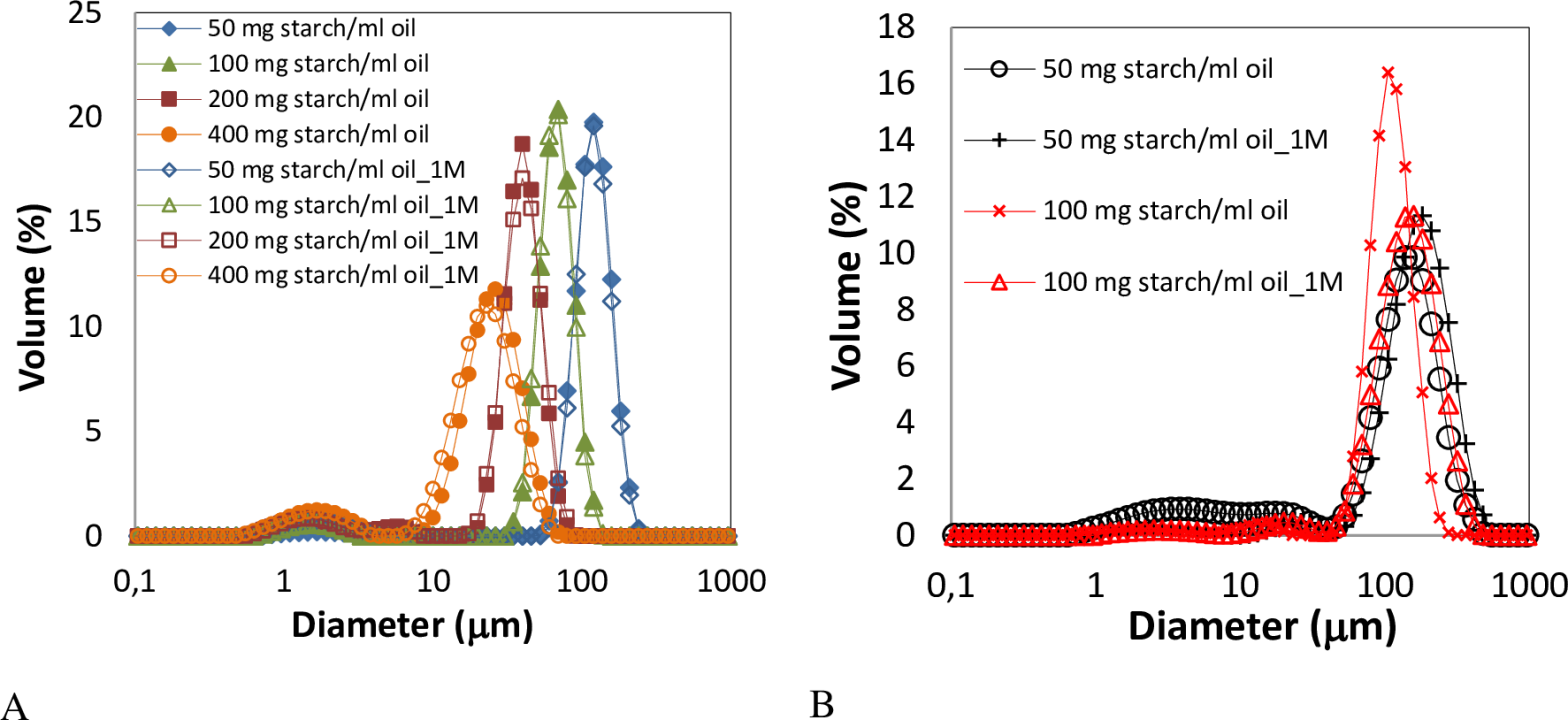
Droplet size distributions of emulsions with 33% (w/w) oil content prepared at different OSA-modified starch concentrations. A) Emulsions stabilized using granules. B) Emulsions stabilized using starch in solution. Droplet size distributions were measured after preparation and after 1 month under storage (1M).

Pickering emulsions prepared with 33% (w/w) oil content showed extreme stability. Mean volume diameters (D_[4,3]_)_,_ and droplet size distributions, did not change after one month of storage at room temperature (Fig 4A). For emulsions prepared with starch in solution (Fig 4B) larger sizes than with Pickering emulsions were obtained besides large differences with time in D_[4,3]_, mode values, and size distributions. As an example micrograph images obtained of both type of emulsions prepared at 100 mg starch/ ml oil are shown in Fig 2C and 2D.

In the latter case, it was only possible to produce emulsions at starch concentrations of 50 and 100 mg per ml of oil since at higher concentrations high viscosity solutions, like a paste, were obtained from autoclave and some aggregates started to appear under microscope just after starch dissolution. These could be attributed to the fact that starch present in solution was not fully dissolved or that due to high starch concentration retrogradation started to occur.

The term retrogradation is used to describe the changes that occur upon cooling and storage in starch after gelatinization, from an initially amorphous state to a more ordered or crystalline state [35,36]. These changes are due to the re-association of starch chains as double helices, and variably ordered semi-crystalline arrays of these helices. Short-term development of crystallinity in starch gels is attributed to the gelation and crystallization of the amylose fraction while the long-term changes that occur during storage of starch gels have been attributed to the amylopectin fraction. It has been reported that the retrogradation was more evident at higher starch concentration and longer storage time [37].

**Fig 5:**
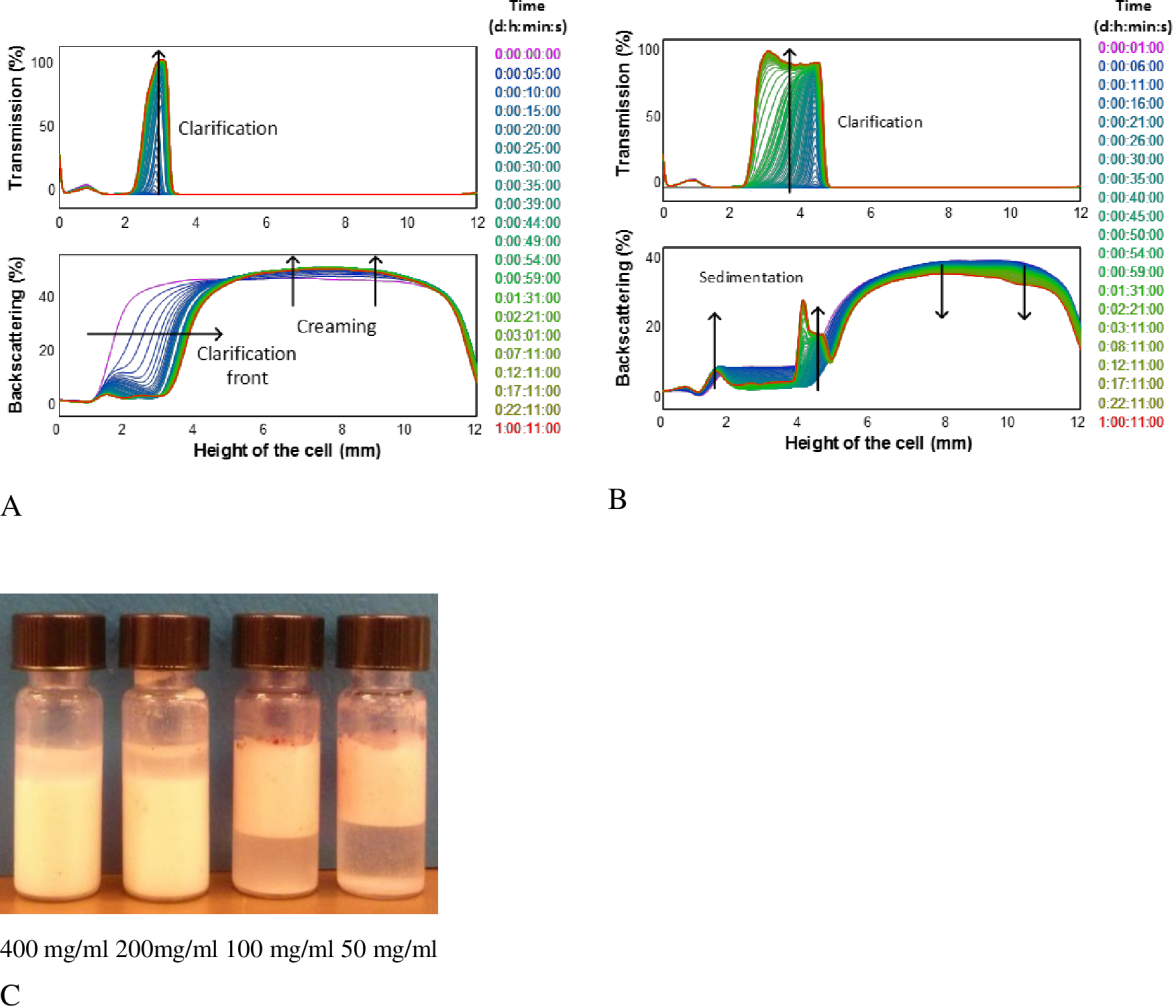
Kinetic ΔBS profiles of emulsions with 33% (w/w) oil content at different OSA-modified starch granules concentrations along the height of the cell. A) Emulsions prepared at concentration of 100 mg starch/ml oil. B) Emulsions prepared at a concentration of 400 mg starch/ml oil. C) Turbiscan glass cells showing the emulsions aspect after the monitoring time. Note: Oil red EGN was added to visualize the eventual presence of free oil.

Fig 5 show the kinetic ΔBS profiles of 33% (w/w) oil Pickering emulsions prepared at different OSA-modified starch granules concentrations. Stability increased with starch content, and higher emulsions index (EI) was obtained compared to emulsions prepared with 7% (w/w) oil content, according to previous studies. No transmitted light was observed for emulsions prepared with 200 and 400 mg starch per ml oil along the height of the cell which means that EI was close to 1, *i*.*e*., the emulsion phase nearly occupied the whole sample (Fig 5C) as it had been reported for emulsions formulated with the same oil content [9].

In the case of emulsion prepared with the lowest starch concentration (50 mg per ml of oil) small sediment appeared at the bottom caused by the unadsorbed starch granules. Similar behavior was previously reported when Pickering emulsions were prepared with several starch types at the same oil content [11].

Fig 5A shows BS and TS profiles of an emulsion stabilized with 100 mg starch/ml oil. A clarification process takes place at the bottom as can be observed in Fig 5C but with no changes in droplet size. The same trend was observed at 50 mg starch/ml oil. Fig 5B shows the BS profile at the 400 mg starch/ml oil. BS does not change all along the height of the cell which is an indication of the extreme stability of these Pickering emulsions. It must be pointed out that last measurement was taken after one month storage. This was confirmed when no color was observed with red oil soluble dye addition at that time (Fig 5C).

**Fig 6:**
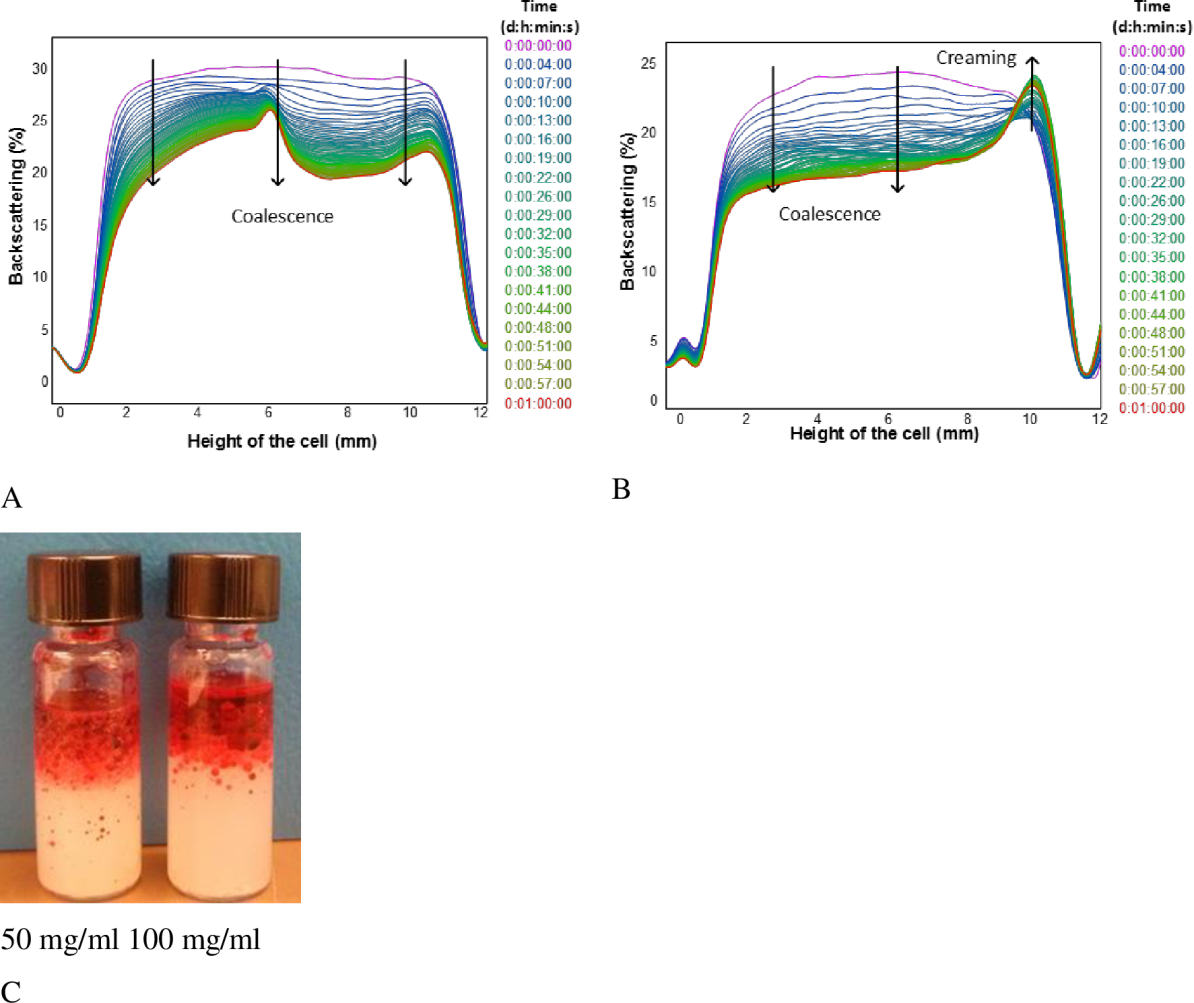
Kinetic ΔBS profiles of 33% (w/w) oil emulsions prepared at different OSA-modified starch concentrations on the dissolved state along the whole height of the cell. A) Emulsions stabilized using 50 mg starch/ml oil. B) Emulsions stabilized using 100 mg starch/ml oil. C) Turbiscan glass cells showing the emulsions aspect after the monitoring time. Note: Oil red EGN was added to visualize the eventual presence of free oil.

As it was aforementioned, from PSD results, emulsions stabilized with OSA-modified starch in the dissolved state showed high instability as observed in Fig 6 where the corresponding BS profiles are depicted. In both cases coalescence was observed and profiles were similar to the ones obtained at 7% oil. Considerable changes in BS (33–40%), and therefore in droplet diameters (35–43%) occurred, although they were lower compared to 7% (w/w) oil emulsions (Table 1). TSI also exhibited lower values in the range 6–7%.

A slight creaming was noticed at the top of the cell, Fig 6B, caused by droplets migration promoted by density differences between oil droplets and continuous phase. This instability is illustrated in Fig 6C where the presence of free oil is also evident. Turbiscan results agree with PSD results and micrograph images.

### Emulsions formulated with a combination of starch granules and dissolved starch

**Fig 7:**
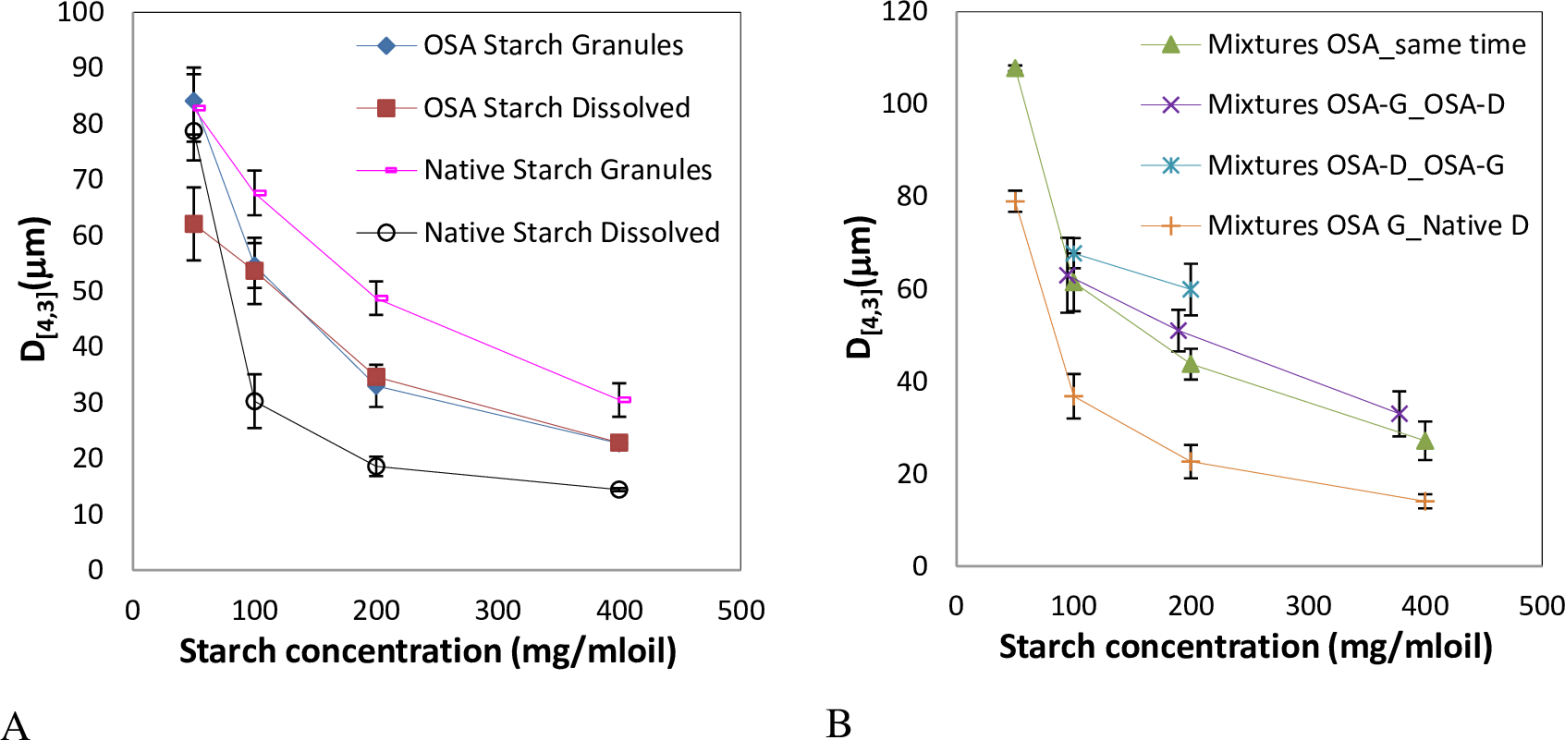
Mean volume diameter, D_[4,3]_, of emulsions prepared with 7% (w/w) oil content at different OSA-modified and native starch concentrations. A) Emulsions stabilized with both types of starches in granules and on the dissolved state. B) Emulsions stabilized with mixtures of both; G:granules; D:dissolved.

Emulsions with 7% (w/w) oil content (Fig 7) were prepared combining OSA-modified starch as granules or in solution in a ratio 1:1 to evaluate the effect of different orders of addition of emulsifier. Mean droplet sizes were larger compared to emulsions prepared individually either with starch granules or in solution (Fig 7A). Particle size distribution on mixed systems containing both fat droplets and starch granules should be treated with caution since they have different refractive indices, and starch granules are non-spherical.

**Fig 8:**
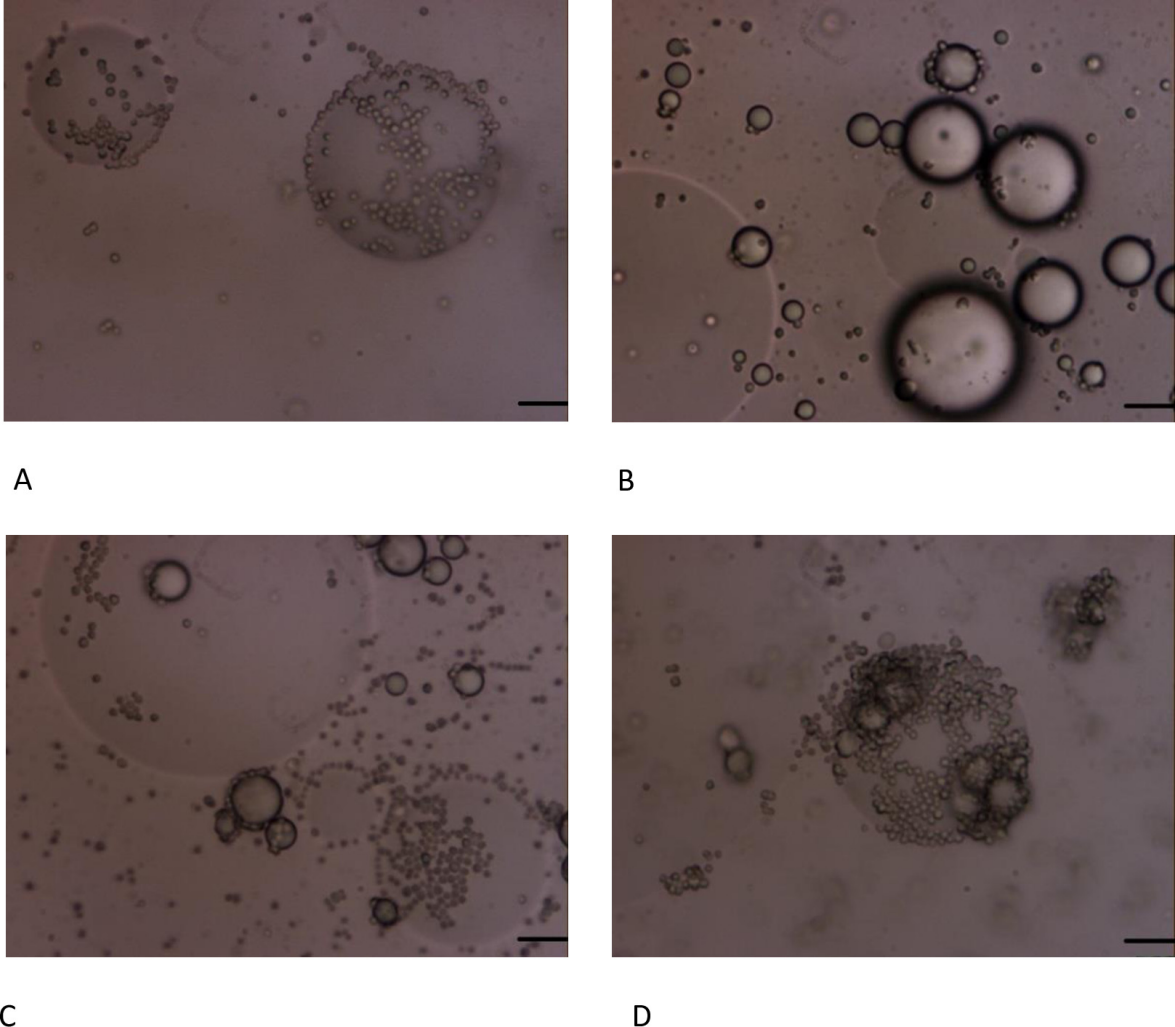
Micrographs of emulsions stabilized by mixtures of OSA-modified starch in the form of granules and on the dissolved state adding both at the same time at different concentrations. A) 50 mg starch/ml oil. B) 100 mg starch/ml oil. C) 200 mg starch/ml oil. D) 400 mg starch/ ml oil. E) Adding first granules and then starch dissolved at 200 mg starch/ ml oil. Scale bar = 10 μm.

Emulsions formulated using a combination of OSA-modified starch granules and OSA starch dissolved (mixtures) were unstable and coalescence was clearly observed under the microscope (Fig 8) and oil soluble dye addition revealed the presence of free oil in all cases (S1 Fig). Moreover, micrographs indicate a high proportion of starch granules in the continuous phase, instead of on droplets surface, according to S1 Fig. Even in the case of emulsions stabilized first with OSA-modified granules on the droplets surface these were later replaced by OSA starch in solution. This indicates that OSA-modified starch is more surface active on the dissolved state than in the granular form, although the emulsion stability is considerable worse.

Microscope images shown in. Fig 8A and 8B correspond to emulsion droplets prepared by adding simultaneously OSA-modified starch in granules and in solution at different concentrations (200 and 400 mg/ml respectively). It seems that for emulsions prepared at lower starch concentrations more granules were adsorbed on the droplet surface. These could be related to the viscosity of the external phase which is higher at higher starch concentrations. Fig 8C and 8D show emulsion droplets prepared at 200 mg starch/ml oil adding first starch granules and then starch in solution. All tested samples were similar under the microscope regarding coalescence and presence of free oil. Therefore, no significant differences were observed in aspect or mean size regarding the different addition sequence for the same OSA starch concentration (S1 Fig).

Sedimentation of unadsorbed granules was observed at the bottom of BS profiles, while a clarification process was observed in the middle of the cell of the TS profiles (results not shown).

Finally, a set of experiments was performed to prepare emulsions stabilized by native starch, both in the granular form and in solution, and also by combining OSA-modified starch granules with native starch dissolved simultaneously.

Emulsions stabilized by native starch granules lead to larger sizes compared to emulsions stabilized by the OSA-modified starch granules, being in the range 30–82 μm (Fig 7A) according to observation in previous results [17].

The opposite effect was obtained with the native starch dissolved leading to lower mean droplet sizes in the range 14–78 μm. Similar sizes were obtained when emulsions were stabilized by mixtures of OSA-modified starch granules and native starch dissolved. Micrographs also revealed the presence of granules in the continuous phase which means that the native starch in the dissolved state was surprisingly more surface active than the OSA-modified granules. However, the stability of these of emulsions was rather poor, coalescence took place quickly and presence of free oil at the top of the samples was readily observed (S1 Fig).

Moreover, emulsions stabilized by native starch in solution showed larger variations in stability (ΔBS, TSI) and size (Δd) were obtained with Turbiscan Lab (Table 1), compared to emulsions stabilized by OSA-modified starch. Variations up to 400% were obtained in D_[4,3]_ values after one month storage. These differences between the two types of starches were also reflected in emulsions stabilized by granules, according to previous results [17]. Emulsions stabilized by native starch granules exhibited variations in D_[4,3]_ values after one month under storage, up to 26%.

The adsorption at emulsions droplets of OSA-modified potato and barely starches was studied by other authors^20^. Results indicated that there was an influence of both of kinetic factors during emulsion formation and the physical-chemical properties of the hydrophobically modified starch, such as the degree of substitution, the molar mass, and radius. It was reported that adsorption of larger molecules was favored by mass transport to the interface in turbulent flow fields and also that these larger molecules will have higher substituent density and adsorption energy than smaller ones [21]. Taking into account that the same trend was found for experiments carried out with modified and native granules in presence of OSA-starch molecularly dissolved, the state of the starch in the external continuous phase (i.e. as granules or dissolved) during the emulsification process could affect the hydrodynamics and mass transport rates of the adsorption process at the interface. Interfacial tension is a fundamental thermodynamic property that affects directly these two factors. It also affects the emulsifying capacity and the tendency for the phases to separate and therefore it could explain the differences observed in emulsions stability. However, interfacial tension also affects the droplets size and similar droplets sizes were obtain when emulsions were prepared by OSA-starch in both states. Therefore, further research should be undertaken to draw a definite conclusion in this respect.

## Conclusions

Results revealed that it is possible to create an emulsion with OSA-modified starch isolated from Quinoa as the sole emulsifier.

Similar droplet sizes were obtained when emulsions were prepared at 7% (w/w) oil content and stabilized using OSA-modified starch in the granular form or on the dissolved state but large differences were observed regarding their stability. Pickering emulsions kept their droplet size constant and were stable against coalescence and other destabilization processes, while emulsions formulated with OSA-modified starch in solution exhibited high instability shown as a clear coalescence process in Turbiscan Lab. Higher emulsions index were obtained at higher oil content for both types of emulsions.

Emulsions stabilized by a combination of OSA-modified starch in granular form and in solution with a 7% (w/w) oil content had larger mean droplet sizes and no significant differences were obtained with respect to the order of addition. All emulsions containing mixtures of granules and dissolved starch were unstable due to coalescence and the presence of free oil was clearly observed in all cases. The same trend was also observed when emulsions were prepared by combining OSA-modified granules with native starch in solution.

The degree of surface coverage of starch granules was much lower in presence of starch in solution. This is an indication that OSA-starch is more surface active in the dissolved state than in granular form, although it led to unstable systems compared to starch granule stabilized Pickering emulsions, which were found to be extremely stable.

## Supporting Information

S1 Fig Turbiscan glass cells containing the emulsions stabilized with mixtures of starch granules and starch dissolved prepared at different order of addition. A) Mixtures of OSA-modified starch in the form of granules and on the dissolved state added at the same time. B) Added first in the form of granules. C) Added first on the dissolved state. D) Mixtures of OSA-modified granules and native starch on the dissolved state added at the same time. Note: Oil red EGN was added to visualize the eventual presence of free oil.
(TIF)
