# Supplementary figures and images for: Comparative Emulsifying Properties of Octenyl Succinic Anhydride (OSA)-Modified Starch: Granular Form vs Dissolved State
Source: PLoS One. 2016 Aug 1;11(8):e0160140. doi: 10.1371/journal.pone.0160140 (PMC4968836; doi:10.1371/journal.pone.0160140)

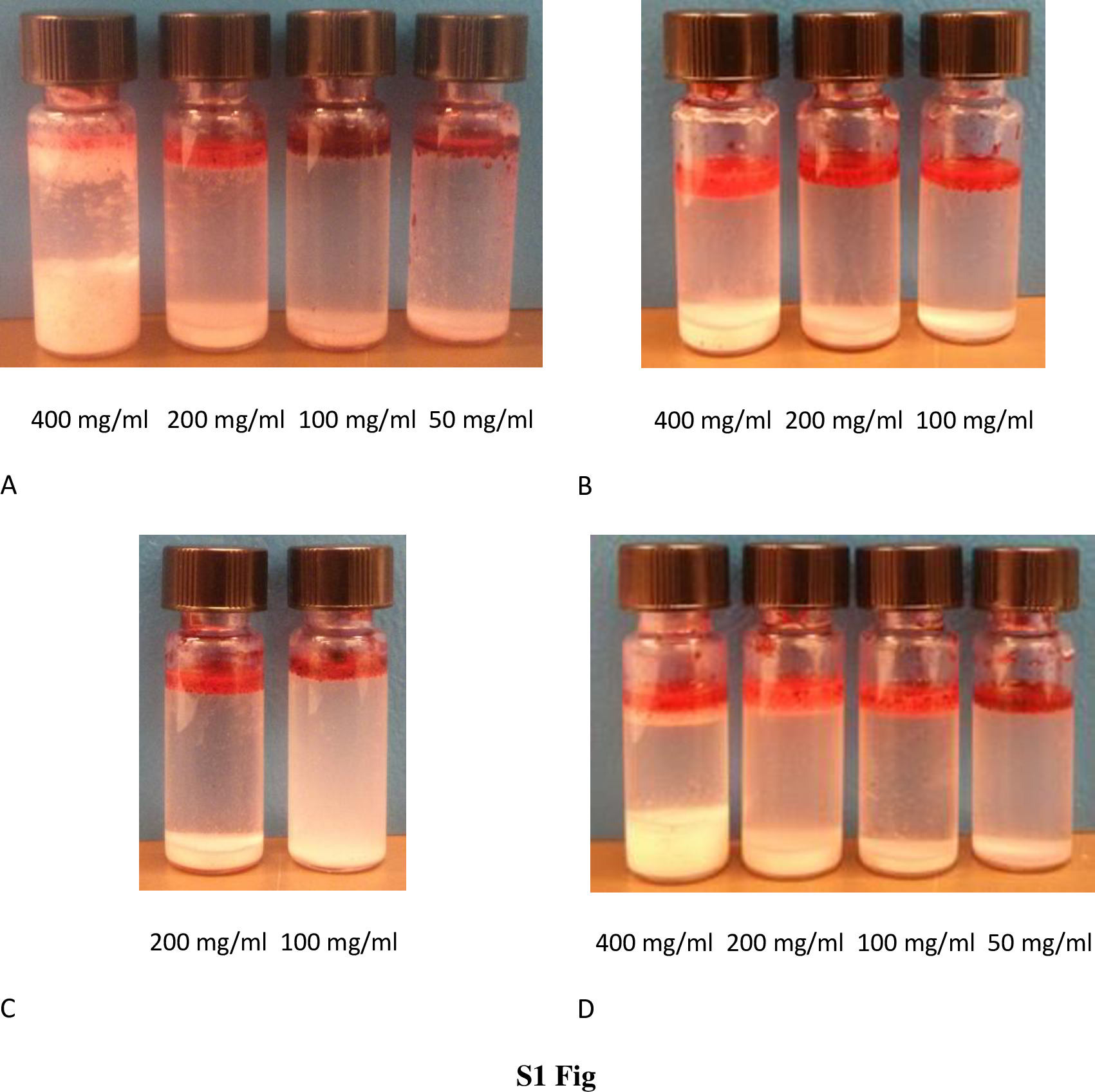

Supplement: S1 Fig — A) Mixtures of OSA-modified starch in the form of granules and on the dissolved state added at the same time. B) Added first in the form of granules. C) Added first on the dissolved state. D) Mixtures of OSA-modified granules and native starch on the dissolved state added at the same time. Note: Oil red EGN was added to visualize the eventual presence of free oil. (TIF) [file pone.0160140.s001.tif]
